# Advances in the Treatment of Cardiac Amyloidosis

**DOI:** 10.1007/s11864-020-00738-8

**Published:** 2020-04-23

**Authors:** Ariane Vieira Scarlatelli Macedo, Pedro Vellosa Schwartzmann, Breno Moreno de Gusmão, Marcelo Dantas Tavares de Melo, Otávio Rizzi Coelho-Filho

**Affiliations:** 1 Department of Cardiology, Irmandade da Santa Casa de São Paulo, Rua Mato Grosso 306, cj1507, Higienópolis, SP Brazil; 2 Cardiology Unit, Unimed Hospital, Ribeirão Preto, Brazil; 3 Department of Onco-Hematology, Beneficência Portuguesa Hospital, São Paulo, Brazil; 4 Department of Internal Medicine, Federal University of Paraíba, João Pessoa, Brazil; 5 Department of Internal Medicine, Discipline of Cardiology, Faculty of Medical Science, State University of Campinas, Campinas, Brazil

**Keywords:** Amyloidosis, Restrictive cardiomyopathy, Transthyretin, Light chain, Amyloid, Heart failure

## Abstract

Cardiac amyloidosis is associated with a high mortality rate, a long delay between the first signs and the diagnosis but a short interval between diagnosis and death. This scenario has changed recently due to improved disease awareness among doctors and significant progress in diagnosis thanks to multimodal imaging and a multidisciplinary approach. Therefore, during the last few years, we have had access to specific therapies for those patients. Those therapies are quite different depending on the type of amyloidosis, but there has been real progress. Systemic light chain amyloidosis (AL) with cardiac involvement is the most common form of cardiac amyloidosis. The severity of heart disease dictates the prognosis in AL amyloidosis. Advances in chemotherapy and immunotherapy that suppress light chain production have improved the outcomes. These recent improvements in survival rates have enabled therapies such as implanted cardiac defibrillators and heart transplantation that were usually not indicated for patients with advanced light chain amyloid cardiomyopathy to now be applied in selected patients. For transthyretin amyloidosis (ATTR), the second most common form of amyloidosis with cardiac involvement, there is also significant progress in treatment. Until recently, we had no specific therapy for ATTR cardiomyopathy (ATTR-CM), though now disease-modifying therapies are available. Therapies that stabilize transthyretin, such as tafamidis, have been shown to improve outcomes for patients with ATTR-CM. Modern treatments that stop the synthesis of TTR through gene silencing, such as patisiran and inotersen, have shown positive results for patients with TTR amyloidosis. Significant progress has been made in the treatment of amyloid cardiomyopathy, and hopefully, we will see even more progress with the spread of those treatments. We now can be optimistic about patients with this disease.

## Introduction

Cardiac amyloidosis (CA) is a systemic disease caused by the extracellular deposition of insoluble amyloid fibrils in the heart [1]. The clinical outcome depends on the extent of tissue involvement and the type of deposited amyloid fibrils. CA should be suspected in patients presenting with HF with preserved ejection fraction, inexplicable left ventricular hypertrophy, and systemic organ involvement, such as neuropathy, anemia, kidney dysfunction, bleeding and thrombosis, dysautonomia, and atrioventricular conduction disturbances [2, 3]. Only 20% have predominant cardiac symptoms, isolated cardiac involvement occurs in less than 5% of cases, and the vast majority have involvement of more than one organ [3]. There are two main types of CA, which correspond to the majority of cases: transthyretin (ATTR) and light chain (AL) cardiac amyloidosis. Amyloid fibrils in AL are composed of monoclonal immunoglobulin light chains and are generally associated with cellular plasma disorders, such as multiple myeloma or other B cell dyscrasias. In ATTR amyloidosis, they come from the transthyretin protein produced in the liver. ATTR amyloidosis more frequently comes from wild-type protein due to age-related misfolding (ATTRwt) and less often from misfolding of variant TTR in patients with a mutation in the TTR gene (ATTRh) [4].

**Fig. 1:**
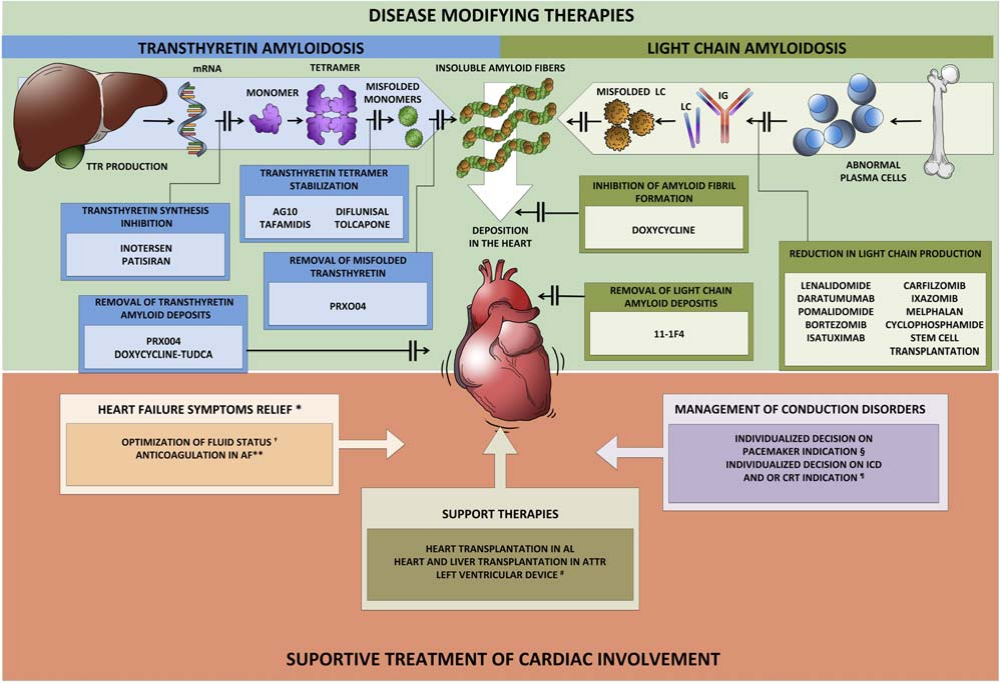
Treatment of cardiac amyloidosis. The management of patients with CA includes a comprehensive approach to administer supportive care as well as the specific treatment regarding the subtype of amyloidosis. The specific treatment for CA interrupts specific steps of amyloidogenesis, such as light chain or transthyretin protein synthesis, formation of amyloidogenic intermediates, or amyloid fibril aggregation. Others try to remove amyloid deposits in the tissue. *The utilization of traditional HF treatment including beta-blockers, ACE inhibitors, or angiotensin-receptor blockers appears to be less effective in these patients and should not be used routinely and perhaps be avoided in selected individuals **Anticoagulation is recommended in all patients with AF and cardiac amyloidosis, and the CHADS-VASC does not apply to that decision. Likewise, anticoagulation should be considered even in patients with sinus rhythm and enlarged atrium due to a high risk of left atrial thrombus promoting atrial dilatation. † Loop diuretics are recommended for fluid overload management. Have caution to avoid preload reduction and hypotension. § There are no current guidelines for the indication or optimal timing of prophylactic pacemaker implantation. # LVAD is feasible in very selected patients. ¶ The role of ICD in cardiac amyloidosis is not well established, and there are few data about CRT. mRNA micro RNA, TTR transthyretin, LC light chain, IG immunoglobulin, ICD implantable cardio defibrillator, CRT cardiac resynchronization therapy, AF atrial fibrillation.

Cardiac amyloidosis has recently gained attention from the medical community for several reasons. First, contemporary cardiac imaging methods have improved, facilitating the diagnosis of CA. The precise differentiation between these two types of CA has important prognostic and therapeutic implications, representing a clinical and imaging challenge. Second, until recently, we had no specific treatment for amyloid cardiomyopathy, but disease-modifying therapies are now available. Most of these new medical advances interrupt specific steps of amyloidogenesis, such as light chain or transthyretin protein synthesis, formation of amyloidogenic intermediates, or amyloid fibril aggregation. Others try to remove amyloid deposits in the tissue with a monoclonal antibody (Fig. 1). These advances represent an essential step forward in the treatment of patients with cardiac amyloidosis and increase the urgency to diagnose it at an early stage, to identify who may benefit from these life-saving therapies.

## When to suspect and how to diagnose cardiac amyloidosis

While CA classically presents with clinical signs of restrictive cardiomyopathy, in daily practice, a restrictive pattern is present in fewer than half of the patients with CA. [5]. Although both TTR and AL CA share similar cardiac functional and morphological characteristics when multimodal imaging techniques are appropriately combined with laboratory and genetic tests, identification of specifying types of CA improves. We recommend using multimodal cardiac imaging, including echocardiogram, cardiac MRI, and nuclear imaging, to differentiate CA, AL, and TTR. The accurate identification of the amyloid subtype is a cornerstone step because the light chain and transthyretin kinds have different prognoses and treatments. If untreated, survival ranges from less than six months for AL CA to three to five years for TTR [6, 7].

While biopsy is a valuable method for obtaining tissue to confirm amyloid infiltration, Congo red staining, which identifies amyloid infiltration as typical apple-green birefringence using polarized light, does not differentiate between the two relevant types of CA. Although heart biopsy is more likely to reveal amyloid deposits in cases with cardiac involvement, tissue may be obtained from other sites, such as abdominal fat, bone marrow, and kidney [8]. In this setting, immunohistochemistry and mass spectrometry can be used. Fat aspiration is a simpler procedure than endomyocardial biopsy. Although its performance is limited in TTR CA, it can be appropriate in AL, as 84% of patients have a positive result [9].

Compelling evidence demonstrates that noninvasive imaging techniques can effectively diagnose TTR CA (Table 1). The echocardiogram is the first-line cardiac imaging method. Particularly in the early stage, it lacks specificity to precisely distinguish amyloid from nonamyloid infiltrative or hypertrophic heart diseases. The classical findings are biatrial enlargement, valvular and interatrial thickening, pleural and pericardial effusion, and biventricular hypertrophy with a bright and sparkling appearance with preserved left ventricular ejection fraction associated with a restrictive pattern with diastolic dysfunction [10]. However, most of these findings are usually found in an advanced stage of disease and are also nonspecific to CA [11]. The presence of a small A wave on mitral inflow Doppler, particularly in the absence of other features of restrictive LV filling, is a clue to identify atrial dysfunction. Cardiac magnetic resonance (CMR) is recognized for its ability to provide gold-standard morphological and functional assessment of the heart. CMR can also provide tissue characterization using multiple sequences, which typically include precontrast T2 imaging for edema and inflation, perfusion for microcirculation assessment, late gadolinium enhanced (LGE) for scar and fibrosis, and T1 mapping (pre- and postcontrast administration) for native T1 and extracellular volume (ECV). Characteristically, patients with CA demonstrate a nonischemic heterogeneous LGE pattern, ranging from transmural or subendocardial to patchy focal LGE, commonly in association with suboptimal myocardial nulling [12]. A pattern of LGE including global subendocardial, transmural, and patchy LGE is very suggestive of CA, with high sensitivity (86%) and specificity (92%) [13]. Although LGE is more common in patients with TTR CA, this finding should not be used to differentiate between subtypes [14]. Recently, the presence of LGE has been shown to be a robust marker for mortality in both LC and TTR CA. Cardiac scintigraphy with bone tracers using a variety of agents (Tc-99m PYP/DPD/HMDP) has revolutionized the TTR CA diagnostic approach characterizing TTR amyloid deposits in the myocardium [15] (Table 1). No plasma or urinary biomarker is available for the diagnosis of TTR CA [11]. Nonetheless, a compound of unusually high plasma levels of N-terminal pro–B-type natriuretic peptide (NT-proBNP) and high troponin levels in a patient with a clinical phenotype of CA should indicate a diagnostic workup. One recent study showed that NT-proBNP is a biomarker that can be highly elevated in ATTRh amyloidosis, especially among asymptomatic carriers of a *TTR* gene mutation or patients with neurological symptoms only [16]. For ATTR-CA, cardiac biomarkers have also recently been used for staging and prognostic stratification. Different staging systems for ATTR-CA have been proposed: one that includes NT-proBNP (> 3000 pg/mL) and troponin T (> 0.05 ng/mL) [17] and another that includes NT-ProBNP and estimated glomerular filtration rate (< 45 mL/min) [18]. Cardiac biomarkers such as natriuretic peptides and cardiac troponins are well-established biomarkers to assess risk and to evaluate response to treatment in patients with AL amyloidosis [19]. Nevertheless, data in AL amyloidosis does not apply to ATTR amyloidosis due to biological differences between the two diseases [19].

**Table 1:** Noninvasive imaging techniques and features in cardiac amyloidosis

Echocardiogram	• Classical findings: biatrial enlargement, valvular and interatrial thickening, pleural and pericardial effusion, biventricular hypertrophy with a bright and sparkling appearance with preserved left ventricular ejection fraction, and a restrictive pattern with diastolic dysfunction. • A regional pattern of strain with severe impairment of strains at the middle and basal segments and relative apical sparing of longitudinal strain [20]. • Myocardial deformation analysis, identifying changes in its measurement on 2-dimensional speckle tracking imaging with a high prevalent rate (93 to 100%) [21]. It has been observed that this strain pattern, known as apical sparing or “cherry on top,” is not specific to CA, since it is also present in other conditions such as aortic stenosis, cardiotoxicity, and dilated cardiomyopathy. • The ejection fraction strain ratio (a ratio of LV ejection fraction/global longitudinal strain > 4.1) improves its accuracy [22].
Cardiac Magnetic Resonance (CMR)	• Provides tissue characterization using multiple sequences. • In patients with atrial fibrillation and in patients with some metallic devices, its application may be limited or restricted. • Typical findings are a nonischemic heterogeneous LGE pattern, ranging from transmural or subendocardial to patchy focal LGE, commonly in association with suboptimal myocardial nulling [12]. • An abnormality in the gadolinium kinetics, also occurring because of systemic amyloid infiltration, drops the blood pool signal to null before the myocardial signal [23]. • A global subendocardial, transmural, and patchy LGE pattern is very suggestive of CA [13]. • More recently, different groups worldwide have investigated the utility of novel CMR metrics based on T1 mapping techniques, with very promising results [23–27].
Nuclear Medicine	• Among the available bone tracers, the most studied has been 99mTc-DPD, which seems to be much more specific to TTR CA compared with AL CA [28, 29]. • 99mTc-DPD is unable to differentiate between inherent and wild-type TTR CA [30]. • Typically, patients with TTR CA have a visual grade ≥ 2, and LA CA patients commonly have no uptake. • It was demonstrated that more than 1 in 5 patients with AL CA have significant uptake of Tc-99m PYP/DPD/HMDP (grades 2 and 3). • The semiquantitative approach using Tc-99m PYP relies on the heart-to-contralateral-lung uptake (H/CL) ratio of > 1.5 at 1 h after tracer administration. This approach was able to precisely differentiate TTR CA from AL CA with high sensitivity (97%) and specificity (100%) [31]. • The semiquantitative approach is also gaining attention because unlike the visual score, it may also provide prognostic information [31].

*99mPYP/DPD/HMDP*, 99m pyrophosphate/dicarboxypropane diphosphonate/hydroxymethylene-diphosphonate

*TTR CA*, transthyretin cardiac amyloidosis

*AL CA*, light chain cardiac amyloidosis

*LGE*, late gadolinium enhancement

*ECV*, extracellular volume

Several diagnostic algorithms have been proposed that incorporate a multimodal imaging approach. The majority of these proposed algorithms start with an investigation to identify classical clinical (TTR gene-positive, aging, low-flow low-gradient aortic stenosis, neuropathy, carpal tunnel syndrome, biceps tendon rupture, lumbar spinal stenosis) and imaging red flags (Table 1). The first step is to rule out AL CA, and depending on the results of the serum-free light chain level and serum and urine immunofixation studies, cardiac scintigraphy using a bone tracer is recommended. Currently, an endomyocardial biopsy is reserved for equivocal imaging findings or in patients with discordant clinical and imaging findings.

## Treatment

### Supportive Care

The management of patients with CA includes a comprehensive approach to administer supportive care as well as the specific treatment for the subtype of amyloidosis. Supportive care is characterized by the treatment of heart failure (HF) symptoms, management of arrhythmias and conduction disorders, and evaluation for heart transplantation [32].

### HF medical therapy

The general approach to patients with CA and HF symptoms should initially consider diet counseling, mainly for sodium restriction and daily weight orientations, to guide diuretic treatment, particularly with loop diuretics to relieve congestion and control symptoms. HF therapy in CA patients is mostly dependent on the monitoring of fluid balance and diuretic usage, and it is suggested that a combination of loop diuretics and an aldosterone antagonist is the most effective approach [33, 34].

Current HF guidelines recommend therapy, including neurohormonal blockade, for patients with HF with reduced ejection fraction irrespective of its etiology [35, 36]. However, regarding amyloidosis etiology, some concerns have been raised regarding potential side effects or detrimental effects in patients receiving neurohormonal blockade. This balance is challenging due to the narrow window between too high and too low filling pressures. Moreover, cardiac output is dependent on heart rate, which added to the tendency of orthostatic hypotension might in part explain the intolerance or ineffectiveness of neurohormonal blockade agents [37]. Additionally, a single-center retrospective cohort study showed reduced survival in patients with TTR CA treated with ACE inhibitors and beta-blockers [32]. Therefore, traditional HF treatment, including beta-blockers, ACE inhibitors, or angiotensin-receptor blockers, appears to be less effective in these patients [33].

In patients with atrial fibrillation and CA, beta-blockers may play a role in rate control, but caution is advised. Nondihydropyridine calcium channel blockers bind avidly to amyloid fibrils and are contraindicated due to the risk of syncope and severe hypotension [33, 37]. Digoxin is usually not recommended in CA because of concerns about an increased risk of toxicity and enhancement of arrhythmogenic effects and sudden death [38]. Recent retrospective data suggested caution with digoxin for rate control in AL amyloid patients with AF. Frequent drug concentration monitoring and renal function control is warranted [39].

### Atrial arrhythmias

Prior studies have shown that cardiac conduction abnormalities and atrial arrhythmias are common among patients with CA [40, 41].

In a retrospective cohort study of 262 patients with CA, 14.5% had AF at baseline, which was more common among patients with ATTRwt, older age, renal dysfunction, greater atrial and systolic left ventricular dimensions, and lower ejection fraction [40]. Surprisingly, and not according to other HF etiologies, the presence of AF was not independently associated with worse all-cause mortality, although it was related to a higher rate of HF hospitalizations [40]. Furthermore, no prognostic differences were observed between paroxysmal and permanent AF in patients with ATTRwt cardiomyopathy [42, 43].

For rhythm management, direct cardioversion has shown similar success rates and AF recurrence rates in patients with vs. without amyloidosis. However, patients with cardiac amyloidosis present more procedure-related complications [43]. Moreover, due to the higher frequency of left atrial thrombus, which was present in 28% of patients with cardiac amyloidosis despite anticoagulation, a transesophageal echocardiogram is recommended before AF cardioversion [44]. Nevertheless, anticoagulation is recommended in all patients with AF and cardiac amyloidosis, and the CHADS-VASC does not apply to that decision. Likewise, anticoagulation should be considered even in patients with sinus rhythm and enlarged atrium due to a high risk of left atrial thrombus [45].

Concerning electrophysiology ablation, a small study has demonstrated that the procedure was generally safe and had acceptable recurrence rates [46]. The patients reported an improvement in the functional class and quality of life. In another study, six of seven patients with CA submitted to ablation experienced a recurrence of arrhythmia in a 2-year follow-up [41]. Therefore, the role of atrial ablation in CA patients demands further investigation.

### Pacemakers

Conduction abnormalities are common in patients with cardiac amyloidosis, although the real prevalence is unknown, and the rates of pacemaker implantation may vary significantly [47]. There are no current guidelines for the indication or optimal timing for prophylactic pacemaker implantation. For patients with life-threatening bradyarrhythmias or symptoms such as syncope and dizziness, the indication should follow current standard guidelines [48•].

### Implantable cardiac defibrillator and cardiac resynchronization therapy

The role of implantable cardiac defibrillator (ICD) in cardiac amyloidosis is not well established. A small study with 46 patients with cardiac amyloidosis due to ATTR and AL has shown that ICD-appropriate therapies were frequent; however, no predictors of ventricular arrhythmias were identified. Moreover, as outcomes were worse in AL patients, the ICD indication is more challenging in that scenario and should be considered on an individual basis, mainly due to the recent survival improvement in those patients. There are several case reports of ICD therapy in AL amyloidosis [49]. Although ICD therapy might play a role in sudden death prevention in high-risk cardiac amyloidosis patients, the efficacy of ICD is controversial because electromechanical dissociation seems to be a significant cause of sudden death in this population [48•]. Current European Guidelines recommend that an ICD be considered in patients with light chain amyloidosis or hereditary transthyretin-associated cardiac amyloidosis and ventricular arrhythmias causing hemodynamic instability who are expected to survive more than 1 year with good functional status (class IIa, level of evidence C) [50].

Regarding cardiac resynchronization therapy (CRT), little evidence supports this indication in cardiac amyloidosis patients. A retrospective analysis of 78 patients with ATTR patients has demonstrated that right ventricular pacing over 40% of the time was related to a worse HF prognosis and deleterious remodeling [51]. For patients who had undergone CRT implantation, surrogate endpoints such as better NYHA functional class, higher left ventricular ejection fraction, and reduced mitral regurgitation were present, but survival or HF hospitalization data are lacking.

### Organ transplantation

Since AL amyloidosis is a systemic condition, patients with severe HF due to AL CA should undergo a thorough investigation to confirm the appropriateness for heart transplantation, excluding individuals with critical extracardiac involvement [52, 53]. One of the most concerning complications in AL CA patients undergoing heart transplantation is the risk of recurrence of amyloidosis after the transplant, causing amyloid deposits in the transplanted heart [54]. Although the experience with heart transplantation in AL CA is limited compared with other etiologies, data from the USA demonstrated that the post-heart transplantation survival is reasonable [55], at 89% for one year and 76% for five years [56].

In ATTR-CA, the liver produces the majority of the TTR, and liver transplantation represents a possible alternative in patients with TTR CA. Liver transplantation has been shown to prevent disease progression in mutant TTR patients, especially modifying the development of peripheral neuropathy in Val30Met patients [57]. While the prognosis of heart transplantation for patients with advanced inherent TTR CA with severe HF is worse compared with other indications, in individuals with no critical extracardiac manifestation, combined heart and liver is an attractive alternative [58, 59].

### Treatment of Light Chain Amyloidosis

The treatment of light chain amyloidosis started with chemotherapy based on melphalan and prednisone [60]. The goal of chemotherapy is the normalization of the involved free light chain (FLC) [61]. Another therapy that emerged was autologous stem cell transplantation (SCT), which produces a rapid response because it can rapidly eradicate the amyloidogenic light chain produced by clonal plasma cell populations [62]. Excellent results with SCT have been reported for patients with cardiac amyloidosis diagnosed before the onset of advanced congestive heart failure. Hematologic and cardiac response rates were 66% and 41%, respectively [63]. Transplant-related mortality rates have decreased from as high as 40% to 4–7% in current studies. Renal and cardiac organ responses and high complete hematologic response rates have been reported after SCT [64].

Induction therapy is a standard component of multiple myeloma (MM) treatment and is offered to AL amyloidosis patients with concurrent MM or with ≥ 10% bone marrow plasma cells, based on the results of one study that suggested that these patients had an outcome similar to patients with coexistent MM and amyloidosis [65]. New drugs for multiple myeloma have been tested in patients with amyloidosis.

### Bortezomib-based regimens

Patients with high tumor burden or who are ineligible for SCT achieved favorable results with combinations including proteasome inhibitors such as cyclophosphamide, bortezomib, and dexamethasone (CyBorD) or bortezomib, melphalan, and dexamethasone, rather than melphalan plus dexamethasone. Rapid responses are seen in a majority of patients, and neuropathy can be dose limiting [66–68]. Randomized trials comparing these regimens with melphalan plus dexamethasone in patients with newly diagnosed AL amyloidosis are ongoing; preliminary results suggest more profound responses with the addition of bortezomib. The most extensive study of CyBorD for the initial treatment of AL amyloidosis was a series of 230 patients from two European referral centers [69]. The overall hematologic response rate was 60% (23% complete). Cardiac and renal responses were seen in 17% and 25%, respectively. After a median follow-up of 25 months, the estimated OS at three years was 55% in the entire population and 52%, 55%, and 19% in patients with Mayo stage II, IIIa (stage III with NT-proBNP ≤ 8500 ng/L), and IIIb (stage III with NT-proBNP > 8500 ng/L), respectively. There were no deaths in patients with stage I disease.

Ixazomib is a new oral proteasome inhibitor; relapse-refractory AL produces a hematologic response in 52% of patients and an organ response in 56% of patients at a dosage of 4 mg by mouth weekly [70].

### The immunomodulatory derivatives

Lenalidomide, pomalidomide, and thalidomide have demonstrated efficacy among patients with relapsed AL amyloidosis but have not been compared with other regimens in this setting.

Thalidomide has been combined with melphalan and dexamethasone in 22 patients, resulting in 8 hematologic and four organ responses [71]. Thalidomide has also been combined with cyclophosphamide and dexamethasone, with a hematologic response rate of 74% and complete response in 21% of patients [72]. Treatment-related toxicity was frequent, and the agent was poorly tolerated. Lenalidomide has also been tested in amyloidosis. In a trial of lenalidomide, melphalan, and dexamethasone with 22 of 25 patients having stage II or III cardiac amyloidoses, the 1-year survival rate was 58%. Organ responses were seen in 8%. Cardiac arrhythmias were seen in 33% [73]. Pomalidomide is a derivative of thalidomide with structural similarity to both thalidomide and lenalidomide. A study of patients previously treated with melphalan, bortezomib, and SCT showed promising results. All patients were evaluable for a hematologic response, with a response rate of 38% [74].

### Monoclonal antibodies

Daratumumab is an anti-CD38 monoclonal antibody used for multiple myeloma. A case report and a retrospective study have described the safety and efficacy of daratumumab in patients with relapsed or refractory AL amyloidosis [75]. In the retrospective study, daratumumab was associated with high rates of hematologic response (76 to 78%), with median times to first response less than three months. Toxicity was similar to that seen in patients with multiple myeloma [76].

Other types of monoclonal antibodies are being developed. In a small trial, the deposition of nontissue amyloid tissue was reduced following the administration of an antibody directed against the amyloid ethical P component (anti-SAP) [77]. A second antibody (NEOD001), targeting the folded protein in AL amyloidosis, demonstrated cardiac responses and renal biomarkers in a prospective study but is not being developed further after a randomized study was discontinued due to poor results [78].

11-1F4 (CAEL-101) is a chimeric monoclonal IgG1 antibody that targets the human Bence-Jones protein, with a stronger affinity for kappa LC than lambda [79, 80].

In a phase Ia/Ib study in patients with relapsed/refractory AL, 62% of patients showed organ response at a median of 2 weeks after starting treatment (NCT02245867) [81]. In patients with AL cardiomyopathy, 11-1F4 led to an improvement in cardiac parameters after 12 weeks of follow-up [82]. A randomized phase II/III trial for newly diagnosed AL patients is planned, including a high-risk patient cohort with NT-proBNP > 8500 ng/L [83].

### Doxycycline

Doxycycline is a tetracycline antibiotic that can also act as an inhibitor of matrix metalloproteinase MMPs. Matrix metalloproteinases (MMPs) and their tissue inhibitors regulate matrix homeostasis in the heart. When the heart is infiltrated by amyloid, a disruption in matrix hemostasis may occur, resulting in myocardial thickening. Some studies have associated inhibition of the MMP pathway with reduction of toxicity of LC in the heart [84].

In a retrospective study with 103 patients with AL CA, the use of doxycycline along with chemotherapy was associated with survival improvement (40 to 82%) and a 3-fold increase in cardiac response to therapy [85]. There are some ongoing clinical trials testing doxycycline along with plasma cell-directed treatment in patients with AL (NCT02207556, NCT03474458, and NCT03401372).

### Treatment of Transthyretin Amyloidosis

New pharmaceutical treatments have emerged to ameliorate TTR amyloidosis, slowing or halting the progression of cardiomyopathy. These specific therapies have affected the outcomes positively and are currently available.

#### Transthyretin tetramer stabilizers

### Tafamidis

Tafamidis is a tetramer stabilizer that binds with high affinity and selectivity to the thyroxine site of TTR, slowing dissociation of TTR tetramers into monomers and preventing aggregation in amyloid fibrils. Tafamidis inhibits nonmutant TTR amyloidogenesis in a dose-dependent manner and stabilizes the two most clinically significant amyloidogenic mutants (V30M and V122I) with similar efficacy [86].

A phase III trial of 441 patients with wild-type and hereditary ATTR-CA tested tafamidis (20 or 80 mg) against placebo [87••]. The pooled tafamidis arms (80 mg and 20 mg) showed a reduction in all-cause mortality (HR 0.70, 95% CI 0.51–0.96) and cardiovascular-related hospitalization (HR 0.68, 95% CI 0.56–0.81). There was also a significant improvement in the 6-min walk test and quality of life, and it was well tolerated. Tafamidis was the first drug approved for the treatment of both wild-type and mutated TTR amyloid cardiomyopathy, in May 2019 [88].

### Diflunisal

In a phase 1 study, the nonsteroidal anti-inflammatory drug diflunisal bound with higher efficacy than thyroxine to the central hormone-binding funnel stabilizing TTR tetramers, thus preventing amyloid fibril formation in vitro [89].

The experience in ATTR-CM is limited to small open-label studies [90–92] where diflunisal (250 mg orally twice daily) was well tolerated, with a low incidence of side effects (thrombocytopenia and renal dysfunction). In one nonrandomized study in ATTR-CM, diflunisal promoted a survival benefit similar to that of tafamidis [92]. Diflunisal might be considered for off-label use in very selected patients with ATTR-CM. We should restrict its use to patients with nonsevere renal dysfunction (glomerular filtration rate of > 45 mL/min/1.73 m^2^), normal platelet count, without signs of fluid overload, or using a high dose of diuretics and with no evidence of recent hemodynamic or renal instability [93••]. The patients should be told to discontinue other nonsteroidal anti-inflammatory agents and to use a proton pump inhibitor. More extensive studies of diflunisal in ATTR-CA are necessary.

### AG10

The selective TTR stabilizer AG-10 is a synthetic small-molecule transthyretin ligand [94]. The primary protein structure of AG10 was identified by a high-throughput screen, then modified with a carboxylic acid group on the 2-fluorophenyl ring to optimize the binding energetics to transthyretin [94]. AG10 binds to wild-type transthyretin with higher affinity than tafamidis or diflunisal. In a phase II randomized, double-blind, placebo-controlled, multicenter study trial, AG10 (400 mg or 800 mg twice daily for 28 days) was well tolerated and induced near-complete stabilization of TTR [95].

There is an ongoing trial evaluating AG10 in the ATTR-CA population. The ATTRIBUTE-CM trial is a phase III prospective, randomized trial (NCT03860935) aiming to evaluate the efficacy and safety of AG10 800 mg in subjects with symptomatic ATTR-CM for a total of 30 months of blinded, placebo-controlled treatment. The final data collection date for the primary outcome measure is planned for April 2023.

### Tolcapone

The catechol-methyltransferase inhibitor tolcapone binds to the thyroxine-binding pocket at the transthyretin dimer-dimer interface with high affinity and strongly inhibits TTR aggregation [96]. It is an FDA-approved Parkinson’s disease therapeutic as an adjunct to levodopa and carbidopa for the treatment of Parkinson’s disease. Tolcapone is presently under investigation in ATTR amyloidosis [93••].

#### Transthyretin synthesis inhibitors

### Patisiran

Patisiran is a second-generation, double-stranded small interfering RNA that blocks the expression of both hereditary and wt TTR [97]. It is modified with 2′-O-methyl ribonucleosides for improved stability and formulated in a lipid nanoparticle. It is delivered to the cytoplasm by endosomal endocytosis of the lipid nanoparticle, where it triggers cellular pathways that control gene expression by RNA interference, leading to a reduction in transthyretin protein levels [98].

In animal models, patisiran reduced transthyretin deposition and facilitated regression of existing transthyretin deposits, with the extent of deposit regression correlating with the level of RNA interference-mediated knockdown [99].

In a phase III trial of patients with hereditary ATTR with polyneuropathy, patisiran (0.3 mg/kg once a day every three weeks for 18 months) significantly improved neuropathy and quality of life in the overall cohort and the subgroup with cardiac involvement (56%; NYHA functional class III/IV were excluded) [100••].

These results led to the approval of patisiran for the treatment of adults with hereditary ATTRh-related polyneuropathy both in the USA and in the European Union.

In a prespecified cardiac subpopulation of the APOLLO trial (56% of the population), patisiran led to a statistically significant reduction in NT-proBNP level, left ventricular wall thickness, improved global longitudinal strain, and increased cardiac output. These findings are suggestive of a cardiac benefit slowing down LV functional deterioration and promoting favorable myocardial remodeling [101].

A new trial to evaluate patisiran in ATTR-CM is ongoing (NCT03997383). The APOLLO-B Phase 3 trial is a randomized, double-blind, placebo-controlled, multicenter study designed to evaluate the efficacy and safety of patisiran in approximately 300 adult patients with ATTR amyloidosis (hereditary or wild type) with cardiomyopathy. Patients will be randomized on a 1:1 basis to receive 0.3 mg/kg patisiran or placebo intravenously administered every three weeks over a 24-month period. After 12 months, all patients will receive patisiran in an open-label treatment period.

### Inotersen

Inotersen is a second-generation 20-O-methoxyethyl–modified antisense oligodeoxynucleotide that lowers hepatic production of both variant and wt TTR [102••]. This molecule is a single-stranded synthetic oligomer that distributes at high levels to the liver, accessing the intracellular space by endosome activity and moving to the nucleus by passive diffusion and active transport [103].

In a unicenter, open-label trial, 15 patients with ATTR-CA (8 ATTRh, 7 ATTRwt) received subcutaneous injections of inotersen (300 mg once a week) for one year. They found a mean peak reduction in TTR concentration of 72% (range from 39 to 91%). Stabilization of cardiac parameters with no significant side effects [102••] was observed.

NEURO TTR was a multicenter, randomized, double-blind, placebo-controlled, phase III trial of patients with hereditary ATTR with polyneuropathy, randomized to inotersen (300 mg weekly) or placebo. After 66 weeks, inotersen was associated with improved neuropathy and quality of life in the overall cohort and the subgroup with cardiomyopathy [104]. Cardiomyopathy was present in 63% of patients, but the study was not powered to measure the effects of inotersen on cardiac disease. Following these results, inotersen has received FDA approval for patients with hereditary ATTR-related polyneuropathy. Thrombocytopenia and glomerulonephritis should be monitored regularly (monitoring of platelet counts weekly and renal function and urinary protein every two weeks) [104].

The efficacy and tolerability of inotersen in ATTR-CM patients is under testing in an ongoing study. The researchers are planning to include 50 patients with both types of ATTR-CM and with NYHA class I-III disease, who will be treated with inotersen (300 mg subcutaneously weekly). They aim to analyze if the drug can slow or stop the progression of TTR amyloid cardiomyopathy and determine the tolerability and safety over 24 months (NCT03702829).

#### Clearance of amyloid deposits

Antibody-mediated removal of amyloid deposits is an area of active development [77]. However, clinical data using this approach have currently resulted in cessation of product development because of futility or toxicity [105]. An antibody that targets TTR residues 89 to 97 (PRX-004; Prothena Biosciences, San Francisco, California) [106] has entered into phase I trials in patients with ATTRh amyloidosis.
